# WDR76 degrades RAS and suppresses cancer stem cell activation in colorectal cancer

**DOI:** 10.1186/s12964-019-0403-x

**Published:** 2019-07-30

**Authors:** Eun Ji Ro, Yong-Hee Cho, Woo-Jeong Jeong, Jong-Chan Park, Do Sik Min, Kang-Yell Choi

**Affiliations:** 1 Translational Research Center for Protein Function Control, Yonsei University, Seoul, Korea; 2 Department of Biotechnology, College of Life Science and Biotechnology, Yonsei University, Seoul, Korea; 3 CK Biotechnolgy Inc, Building 117, 50 Yonsei Ro, Seodemun-Gu, Seoul, Korea; 4 Department of Molecular Biology, College of Natural Science, Pusan National University, Pusan, Korea

**Keywords:** Colorectal Cancer, RAS stability, WDR76, Cancer stem cell, Tumor suppressor

## Abstract

**Background:**

Stabilization of RAS is a key event for the hyper-activation of Wnt/β-catenin signaling and activation of cancer stem cell (CSC) in colorectal cancer (CRC). WD Repeat protein 76 (WDR76) mediates the polyubiquitination-dependent degradation of RAS in hepatocellular carcinoma (HCC). We investigated whether WDR76 destabilizes RAS and acts as a tumor suppressor inhibiting CSC activation in CRC.

**Methods:**

We generated mice with deletion of *Wdr76* (*Wdr76*^*−/−*^) and crosses of *Wdr76*^*−/−*^ with *Apc*^*Min/+*^ (*Wdr76*^*−/−*^*; Apc*^*Min/+*^) and compared them with wildtype mice (*Wdr76*^*+/+*^) and *Apc*^*Min/+*^ mice (*Wdr76*^*+/+*^*; Apc*^*Min/+*^), respectively. Intestinal crypt lengthening, tumorigenesis and CSC activation were analyzed by histology, immunohistochemistry, and immunoblotting. CRC cell line was engineered to stably express or knockdown WDR76 or control vector and was analyzed after spheroid culture.

**Results:**

*Wdr76*^*−/−*^ mice, with increased Ras level, displayed crypt elongation and hyper-proliferation. *Wdr76*^*−/−*^*; Apc*^*Min/+*^ mice developed more tumors with bigger sizes than *Apc*^*Min/+*^ mice and their tumors showed increased proliferation and CSC activation with elevated RAS and β-catenin levels. In CRC cells, overexpression or knockdown of WDR76 decreased or increased the numbers and sizes of CRC spheroids with inhibition or activation of CSC markers, respectively. In human CRC, lower level of WDR76 was associated with poor patient survival.

**Conclusions:**

In analyses of mice with deletion of *Wdr76* and CRC spheroids, we found that RAS stability plays important roles in tumorigenesis by affecting proliferation and CSC activation. Our results suggest that destabilization of RAS by WDR76 is a potential strategy for targeting malignant CRC involving CSC activation.

**Graphic abstract:**

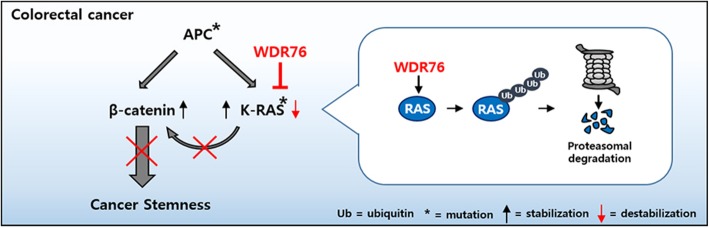

**Electronic supplementary material:**

The online version of this article (10.1186/s12964-019-0403-x) contains supplementary material, which is available to authorized users.

## Background

Colorectal cancer (CRC) is a stem cell disease that occurs when an intestinal stem cell (ISC) escapes regulation and gives rise to a cancer stem cell (CSC) [1–3]. CSCs drive initiation, progression and subsequent metastasis of CRC [4]. Thus, identification and targeting of factors involved in CSC activation has emerged as a promising strategy for therapeutic intervention in CRC [5–7]. Despite recent advances in the identification of molecular CSC markers and CSC-activating signaling pathways, it is difficult to selectively target CSCs without affecting normal stem cells; the available markers do not adequately distinguish between CSCs and normal stem cells, and the signaling pathways that activate CSCs also play essential roles in tissue homeostasis and repair [8]. A better understanding of the differences between normal and malignant stem cells may make it possible to specifically target CSCs while avoiding toxicity to normal stem cells [6].

The intestinal crypt stem cell niche provides Wnt and epidermal growth factor (EGF) signals that maintain resident stem cells and can also instruct progenitor cells to revert to a stem cell state when the original stem cells are lost. Similarly, when CSCs are eradicated, transit amplifying (TA) cells and differentiated cells can be reprogrammed into CSCs by the niche through plasticity. Thus, CSCs can always be re-created if the CSC niche with aberrantly activated signaling remains uncontrolled. CRC tumor progression occurs through the acquisition of genetic alterations in signaling pathways, such as the Wnt/β-catenin and EGF/RAS pathways that maintain the self-renewal and proliferation of normal ISCs, respectively. Those pathway alterations result in the autonomous acquisition of aberrant stemness by cancer cells [9].

Wnt/β-catenin signaling sustains ISCs and intestinal homeostasis [8]. *Adenomatous polyposis coli* (*APC)* mutation occurs in as many as 90% of CRCs and causes aberrant activation of Wnt/β-catenin signaling, leading to clonal expansion of ISCs and adenoma formation. *K-RAS* mutation, which occurs in 40–50% of advanced CRCs [10–12], does not by itself result in CSC activation [13, 14]; however, in the presence of *APC* mutation, oncogenic *K-RAS* mutation results in CSC activation involving malignant transformation as well as metastasis occurs by co-presence of *K-RAS* and *APC* mutations, attributed to initial activation of the Wnt/β-catenin signaling and its subsequent strong further activation by stabilization of oncogenic K-RAS by *APC* loss [15]. The regulation of oncogenic K-RAS stability via Wnt/β-catenin signaling is a key event for the crosstalk between the Wnt/β-catenin and RAS signaling pathways [15, 16]. Interaction between β-catenin and RAS acts as a molecular switch for the Wnt/β-catenin signaling-dependent regulation of RAS stability [17]. Nevertheless, the role of RAS stabilization in ISC and CSC activation has not been fully investigated. In addition to the regulation by Wnt/β-catenin signaling, a recent study revealed an alternative route to directly target RAS stability [18].

WDR76 (WD Repeat protein 76) is an E3 ligase that suppresses tumorigenesis of human hepatocellular carcinoma (HCC) cells by destabilizing RAS [19]. WDR76 has been identified as one of the proteins that interacts with H-RAS in human HCC tissues and destabilizes all three major RAS isoforms, H-RAS, K-RAS and N-RAS [19]. Here, based on our observation of increased RAS protein levels in CSC-like population with high expression of CSC markers compared to low CSC counterpart sorted from a human CRC cell line, we investigated the role of WDR76 as a modulator of RAS abundance and CSC activation contributing to tumorigenesis in CRC.

Overexpression of WDR76 in spheroids derived from a human CRC cell line harboring both *APC* and *K-RAS* decreased K-RAS abundance and effectively suppressed CSC activation. Moreover, loss of *Wdr76* increased Ras abundance and exacerbated tumorigenesis in *Apc*^*Min/+*^ mice. Those results implicate WDR76 as a novel tumor suppressor that directly regulates RAS protein stability and CSC activation in CRC. These observations, underscoring the importance of RAS stability in ISC and CSC activities, provide insights into colorectal tumorigenesis and therapeutic development.

## Methods

### Cell culture and reagents

Human CRC cells (SW480, DLD-1 and HCT116) were obtained from the American Type Culture Collection (Manassas, VA). Isogenic human DLD-1 CRC cell lines expressing either wild-type or mutant *K-RAS* (D-WT and D-MT cells, respectively) were provided by B. Vogelstein (Johns Hopkins Oncology Center) [15]. Cells were propagated at 37 °C and 5% CO_2_ in DMEM (Gibco) or RPMI 1640 (Gibco) supplemented with 10% FBS (RMBI) and 1% penicillin-streptomycin (Gibco). Lipofectamine (Invitrogen) was used for plasmid transfection, according to the manufacturer’s instructions. ALLN (25 μg/mL; Sigma-Aldrich) and MG132 (20 μM; AMRESCO) were added to media to inhibit protein degradation.

### Lentivirus production and establishment of stable cell lines

For viral production, HEK293T cells were transfected with pLVX-GFP-Control, pLVX-GFP-WDR76, pLVX-GFP-WDR76ΔNLS, pLKO.1-shControl-GFP, or pLKO.1-shWDR76-GFP, [19] together with the viral packaging psPAX2 and viral envelope pMD2G plasmids at a 2:2:1 ratio. Then, D-WT and D-MT cells were transduced with each lentivirus. Stable cell lines were selected either by Hygromyicn B (Duchefa) or by puromycin (Sigma) treatment for 2 weeks.

### Spheroid culture

1 × 10^4^ cells/ml were seeded with serum-free medium containing DMEM/F12 (Invitrogen) supplemented with B27 (Invitrogen) and 20 ng/ml EGF and 10 ng/ml bFGF (Peprotech) in a 90.00 mm × 15.00 mm petri dish (SPL). Experimental procedures were performed after 5 days of spheroid-forming culture. The number and size of the spheroids were measured using Image J software.

### Immunoblotting

Cells were washed with ice-cold PBS and lysed with radio immunoprecipitation assay (RIPA) buffer [150 mM NaCl, 10 mM Tris pH 7.2, 0.1% sodium dodecyl sulfate (SDS), 1% Triton X-100, 1% sodium deoxycholate, and 5 mM ethylenediaminetetraacetic acid (EDTA)]. Samples of mouse tumor tissues stored in liquid nitrogen were prepared in RIPA buffer and then homogenized. Proteins (10–20 μg) were separated using 10–12% SDS polyacrylamide gel and transferred to a nitrocellulose membrane (Whatman). After blocking with 5% skim milk in tris buffered saline (TBS) containing 0.1% Tween20 (Sigma) for 1 h, the membrane was incubated with the primary antibodies: anti-β-catenin (BD Bioscience), anti-RAS monoclonal (Millipore), anti-K-RAS (Santa Cruz Biotechnology), anti-p-ERK (Cell Signaling Technology), anti-p-AKT (Cell Signaling Technology), anti-PCNA (Santa Cruz Biotechnology), anti-Flag (Cell Signaling Technology), anti-GFP (Santa Cruz Biotechnology), and WDR76 antibody purified as described previously [19]. Horseradish peroxidase-conjugated anti-mouse (Cell Signaling Technology) or anti-rabbit (Bio-Rad) antibodies were used as secondary antibodies. Immunoblots were visualized by enhanced chemiluminescence (Amersham Bioscience) using a luminescent image analyzer (LAS-3000, Fuji Film). Three biological replicates of each cell line were analyzed.

### Immunoprecipitation and ubiquitination assays

D-WT and D-MT cells transfected with plasmids expressing ubiquitin were used for the ubiquitination assay. Cells were washed in ice-cold PBS (Gibco) and lysed with RIPA buffer with N-ethylmaleimide (10 mM; Sigma-Aldrich). The lysates were immunoprecipitated by incubating with K-RAS antibody and protein G agarose beads (Thermo Fisher Scientific) with rotation at 4 °C for 12 h. The beads were washed three times in RIPA buffer. The resulting immune complexes were resolved by SDS-PAGE, and immunoblotting was performed with the indicated antibodies. Three biological replicates were performed.

### RNA isolation and quantitative real-time PCR

Total RNA was prepared from cells using TRIzol (Invitrogen) following the manufacturer’s instructions. Total RNA (2 μg) was reverse transcribed using 200 U reverse transcriptase (Invitrogen) in a 20 μL reaction with M-MLV reverse transcriptase (Invitrogen). PCR amplification and detection of the PCR-amplified gene products were performed with the SYBR Green PCR master mix (Qiagen). Levels of mRNA expression were quantified after normalization to β-actin endogenous control using the ΔCT (difference between cycle thresholds) method. Three biological replicates were performed.

### Flow cytometry analysis

D-MT cells were dissociated into single cells by trypsin-EDTA, fixed with formalin (Sigma), and then incubated with anti-human CD133-PE (Miltenyi Biotec), anti-human CD166-PerCP-eFluor (eBioscience), and anti-human/mouse CD44-APC (eBioscience) and sorted by flow cytometry (BD Bioscience).

### Immunocytochemistry

Spheroids were harvested at day 5 of spheroid culture, fixed with acetone, and embedded in 1% agarose. 1% Agarose blocks were dehydrated and embedded into a paraffin block. Paraffin-embedded spheroid sections (4 μm thick) were rehydrated, permeabilized with 0.1% triton X-100, blocked with 5% bovine serum albumin (BSA), and incubated with the primary antibodies: anti-GFP (Santa-Cruz Biotechnology), anti-panRAS (Millipore), anti-β-catenin (BD Bioscience), anti-CD44 (ProteinTech), anti-CD133 (ABBIOTEC), and anti-CD166 (ABBIOTEC). For secondary antibodies, Alexa Fluor 488 (Life Technologies) or Alex Fluor 555 (Life Technologies) was used. Counterstaining was done with 4′6’-diamidino 2-phenylindole (DAPI; Sigma). Gel/Mount media (Biomeda Corporation) was used for mounting. Immunofluorescent images were captured using confocal microscopy (LSM 700, Carl Zeiss). At least five fields per section were analyzed.

### Animal model and analysis of tumor tissues

All animal experiments were performed in accordance with the Korean Food and Drug Administration guidelines. Protocols were reviewed and approved by the Institutional Animal Care and Use Committee (IACUC) of Yonsei University. C57BL/6 J-*Apc*^*Min/+*^ (*Apc*^*Min/+*^), B6.129P2-Lgr5tm1(cre/ERT2)Cle/J (*Lgr5-EGFP*), and B6.129S-*Kras*^*tm3Tyj*^ (*K-Ras*^*G12D*^*LA2*) mice were obtained from Jackson Laboratory (Bar Harbor, ME) or the National Cancer Institute mouse repository (National Institutes of Health Technology Transfer Center, Bethesda, MD). C57BL/6 J-*Wdr76*^*+/+*^ (*Wdr76*^*+/+*^) and C57BL/6 J-*Wdr76*^*−/−*^ (*Wdr76*^*−/−*^) mice were generated as previously described [19]. Mouse genotyping was performed using genomic DNA extracted from the toe. To control for genetic background effects, sex-matched littermates were used as controls. Immediately after sacrifice, the abdomen of each mouse was cut open longitudinally and cleaned by flushing twice with cold PBS. After vigorous washing, resected tissues were fixed with 10% neutral formaldehyde (Sigma) for 24–48 h at 4 °C. The fixed tissues were embedded in paraffin according to standard procedures. The tumors were classified according to standard World Health Organization histopathological criteria. For immunoblotting analyses, a subset of freshly isolated tissues was snap frozen in liquid nitrogen and stored at − 80 °C. The mean ± standard deviation was reported based on six mice for each group.

### Immunohistochemistry

Paraffin-embedded tissue sections (4 μm thick) were deparaffinized, rehydrated, and autoclaved with 10 mM citrate buffer (pH 6.0) for 15 min. The sections were blocked with 5% BSA and 1% NGS in PBS for 1 h and were incubated with primary antibodies; anti-WDR76 (Novus biologicals), anti-RAS (Millipore), anti-β-catenin (BD Bioscience), anti-PCNA (Santa Cruz Biotechnology), anti-Ki67 (Abcam), anti-GFP (Abcam), anti-CD44 (eBioscience), anti-CD133 (eBioscience), anti-CD166 (ABBIOTEC), anti-Mucin2 (Santa Cruz Biotechnology), or anti-Lysozyme (Santa Cruz Biotechnology); overnight at 4 °C, followed by incubation with secondary antibodies (1:500) for 1 h at room temperature. The antibodies were diluted with PBS containing 1% BSA and 1% NGS. The sections were counterstained with DAPI (Sigma-Aldrich) and mounted in Gel/Mount medium (Biomeda Corporation). The fluorescence signals were visualized using confocal microscopy (LSM700, Carl Zeiss) at excitation wavelengths of 488 nm (Alexa Fluor 488), 543 nm (Alexa Fluor 555) or 405 nm (DAPI). For peroxidase IHC analysis, sections were blocked by 3.42% H_2_O_2_ and incubated with primary antibodies overnight at 4 °C, followed by incubation with biotinylated anti-mouse or anti-rabbit (Dako) secondary antibodies for 1 h at room temperature. The samples were then incubated using an ABC kit (Vector Laboratories) for 1 h, stained with 3, 3′-diaminobenzidine (DAB; Dako) for 3–7 min, and counterstained with Mayer’s hematoxylin (Muto). Signals were analyzed using a bright field microscope (Nikon ECLIPSE 80i). All incubations were conducted in humid chambers. At least three fields per section were analyzed.

### Tissue microarray sample analysis

Tissue microarray (TMA) slides for normal and colon adenocarcinoma (BC05002)) were purchased from US Biomax, and IHC analyses was performed with β-catenin or Ras as described in previous studies [20]. The TMA slides were visualized by microscopy (Eclipase 80i, Nikon). For quantitative analysis, H-Score of each staining was determined by IHC profiler software. H-Score = 3* highly positive population + 2 * Positive population + 1* weak positive population + 0* negative population.

### Bioinformatics analysis

For Kaplan Meier analysis, publicly available TCGA data in the Human Protein Atlas (https://www.proteinatlas.org/pathology) were used. FPKM values of WDR76 were split into high and low groups using percentile combinations of 66–33% (higher than 66% and lower than 33%). Publicly available microarray expression data in the Gene Expression Omnibus (GEO accession GSE21510) were used for gene expression analysis.

### Statistical analyses

All statistical analyses were performed using Microsoft Excel (Microsoft, Redmond, WA) or GraphPad Prism5 Software (GraphPad, La Jolla, CA). Group differences were determined with the Student *t* test. Data were expressed as means with standard deviation. All statistical tests were two-sided, and *P* values less than 0.05 were considered statistically significant.

## Results

### Loss of *Wdr76* increases the Ras protein level and induces crypt elongation

The EGF/mitogen-activated protein kinase (MAPK) pathway and oncogenic *K-Ras* both play roles in the proliferation of Lgr5^+^ ISCs [14, 21]. Furthermore, the abundances of β-catenin and RAS are correlated with each other in human CRC [15, 16]. Accordingly, we found that pan-Ras was stabilized in the intestinal crypt base, where Lgr5^+^ ISCs reside in the murine small intestine (Additional file 1: Figure S1A, B).

**Fig. 1:**
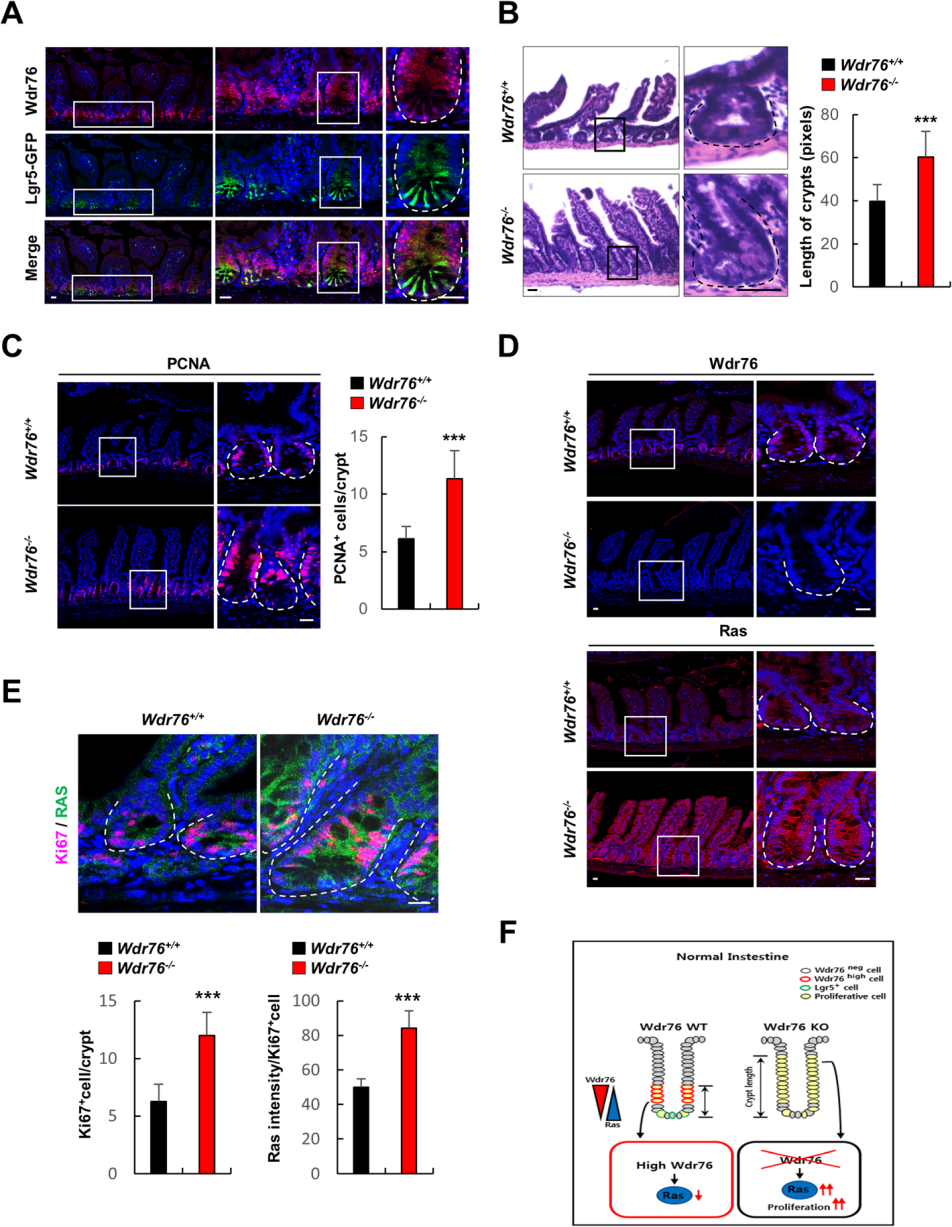
Loss of Wdr76 increases Ras protein level and crypt length in the murine small intestine. **a** Confocal immunofluorescence of Wdr76 and Lgr5-GFP in *Lgr5-EGFP* mouse intestinal sections: Wdr76 (red), Lgr5-GFP (green), and DAPI (blue). **b-e** Analysis of small intestinal sections of 15-week-old *Wdr76*^*+/+*^ and *Wdr76*^*−/−*^ mice **b** H&E stained sections of small intestine from *Wdr76*^*+/+*^ and *Wdr76*^*−/−*^ mice and quantification of the length of small intestinal crypts. **c** Confocal immunofluorescence and quantification of PCNA in small-intestine sections of *Wdr76*^+/+^ and *Wdr76*^−/−^ mice. **d** Confocal immunofluorescence of Wdr76 (red) and Ras (red) in intestinal sections of *Wdr76*^*+/+*^ and *Wdr76*^*−/−*^ mice. **e** Confocal immunofluorescence of Ki67 and Ras in small-intestine sections of *Wdr76*^*+/+*^ and Wdr76^−/−^ mice with quantifications of Ki67^+^ cells in crypts and Ras expression levels in Ki67^+^ crypt cells. **a-e** Boxes indicate the enlarged areas. Crypts are indicated by dotted lines. Scale bars represent 20 μm. All measurements or counts are based on at least 10 crypts per 5 fields of view. *** *p* < 0.001. **f** Schema depicting the effects of *Wdr76* deletion (Wdr76 KO) in normal small intestine

To investigate the role of Ras protein stabilization in the crypt base of the normal small intestine, we examined the expression of the Ras-degrading E3 ligase Wdr76 using *Lgr5-EGFP* mice [1]. Wdr76 is mostly expressed at the crypt side walls, where TA cells reside and the expression levels of Wdr76 and Lgr5 are almost inversely correlated in the crypts (Fig. 1a), suggesting that it may act as a stem cell suppressor. We examined the phenotypes of *Wdr76*-knockout (*Wdr76*^−/−^) mice [19] and found that loss of *Wdr76* did not induce tumor formation in 15-week-old mice; however, it induced significant crypt lengthening and hyper-proliferation (Fig. 1b, c, Additional file 1: Figure S2A), which are characteristics of mice that harbor oncogenic *K-Ras* mutations [13].

WDR76 was previously identified as an E3 ligase linker protein that destabilizes RAS in HCC [19], so we examined whether Wdr76 regulates Ras stability in the small intestine. Deletion of *Wdr76* significantly increased pan-Ras levels along the crypt-villi axis of the small intestine, especially in the crypt side walls (Fig. 1d). These results showed that endogenous Wdr76 restricts Ras abundance by regulating the stability of the Ras protein. The *Wdr76*^−/−^ mice had increased numbers of Ki67-positive proliferative cells, which displayed increased pan-Ras abundance, indicating the importance of Ras stabilization in the proliferation induced by *Wdr76* loss (Fig. 1e). Mucin2 staining indicated that *Wdr76* deficiency also increased the lineage differentiation into goblet cells (Additional file 1: Figure S2B), in agreement with previous results showing the importance of the epidermal growth factor receptor (EGFR)/MAPK pathway in goblet cell differentiation [21].

Our results showed that Wdr76 regulates intestinal homeostasis by restricting Ras protein expression to the cells in the intestinal crypt bottom, where Lgr5^+^ ISCs reside. The deletion of *Wdr76* increases pan-Ras protein abundance and induces crypt elongation, which can develop into precancerous crypt hyperplasia, suggesting that the regulation of Ras protein stability is important in intestinal homeostasis (Fig. 1f).

### Loss of Wdr76 enriches CSCs and enhances tumorigenesis in *Apc*^*Min/+*^ mice

**Fig. 2:**
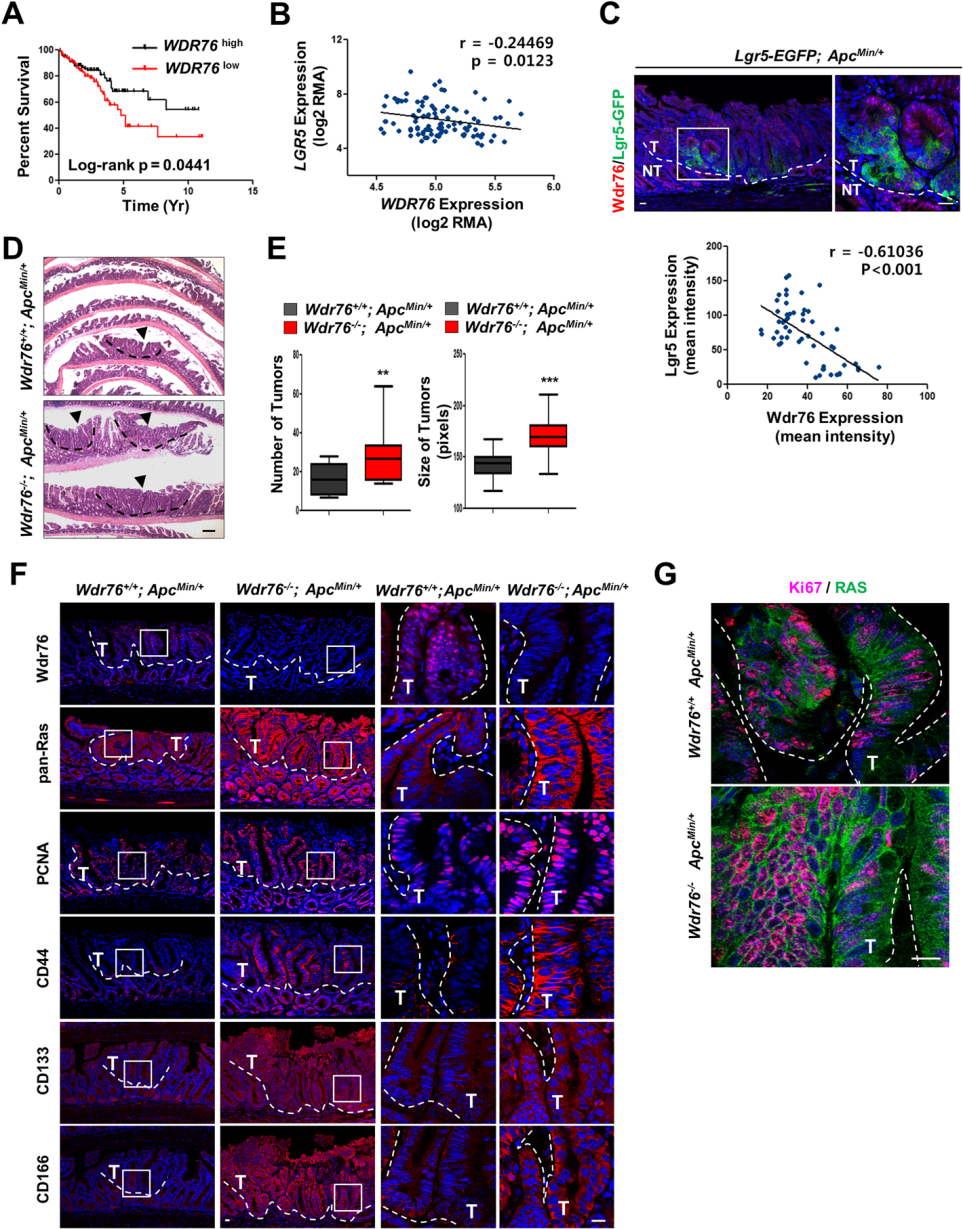
Loss of Wdr76 increases Ras protein levels and exacerbates CRC tumorigenesis. **a** Kaplan-Meier analysis of overall survival in colon cancer patients, classified by *WDR76* expression (WDR76 high and WDR76 low groups are defined as those with FPKM values above the 66th percentile and below the 33rd percentile, respectively). Log-rank (Mantel-Cox) test, *p* = 0.0441. **b** Linear regression curve showing *LGR5* and *WDR76* expression levels in log_2_ in 104 CRC patients (GSE21510). **c** Confocal immunofluorescence of Wdr76 and Lgr5-GFP in *Apc*^*Min/+*^*; Lgr5-EGFP* intestinal sections with linear regression curve showing Lgr5 and Wdr76 expression in tumors. Quantitative expression levels based on at least 10 tumors per 10 fields of view. *** *p* < 0.001. Wdr76 (red), Lgr5-GFP (green), and DAPI (blue). Boxes indicate the enlarged areas. Tumor areas are indicated by dotted lines. T: Tumor; NT: Non-tumor. Scale bars represent 20 μm. **d-g** Analysis of small intestinal sections of 15-week-old *Wdr76*^*+/+*^*; Apc*^*Min/+*^and *Wdr76*^*−/−*^*; Apc*^*Min/+*^ mice **d** H&E stained sections of small intestines from *Wdr76*^*+/+*^*; Apc*^*Min/+*^and *Wdr76*^*−/−*^*; Apc*^*Min/+*^ mice. Tumors are indicated by arrowheads. Scale bars represent 20 μm. **e** Quantification of the total number and size (pixel) of tumors per mouse *(Wdr76*^*+/+*^, *n* = 6; *Wdr76*^*−/−*^, n = 6). **p < 0.01. **f** Confocal immunofluorescence of Wdr76, Ras, PCNA, CD44, CD133, and CD166 in sections of small intestine from *Wdr76*^*+/+*^*; Apc*^*Min/+*^and *Wdr76*^*−/−*^*; Apc*^*Min/+*^ mice. Boxes indicate the enlarged areas. Tumors are indicated by dotted lines. T: Tumor. Scale bars represent 100 μm (left) and 20 μm (right). **g** Confocal immunofluorescence of Ki67 (red) and Ras (green) in tumor sections of *Wdr76*^*+/+*^*; Apc*^*Min/+*^and *Wdr76*^*−/−*^*; Apc*^*Min/+*^ mice. Tumors are indicated by dotted lines. Scale bars represent 20 μm

Given that dysregulation of stem cell activity has been associated with intestinal tumorigenesis, and that CSC activation involving metastasis and chemoresistance results in poor clinical outcomes, we analyzed the prognostic value of WDR76 in CRC patients. Low WDR76 expression was associated with poor prognosis (Fig. 2a). The mRNA expression level of *WDR76* was inversely correlated with the expression level of *LGR5* (Fig. 2b), a relevant CSC marker associated with poor prognosis [22]. Consistent with that, the absence of Wdr76 expression observed in Lgr5-GFP^+^ cells in the normal murine small intestine is also exhibited in tumors derived from crosses between *Lgr5-EGFP* mice and the murine CRC model, *Apc*^*Min/+*^ mice [23]. Quantification of Wdr76 and Lgr5 protein expression in mouse tumors showed that, consistent with the mRNA expression patterns of *WDR76* and *LGR5* in human CRC, the expression levels of Wdr76 and Lgr5 were inversely correlated in murine CRC (Fig. 2c).

To characterize the role of WDR76 in CRC tumorigenesis, we crossed *Wdr76*^*−/−*^ mice with *Apc*^*Min/+*^ mice to generate *Wdr76*^*−/−*^*; Apc*^*Min/+*^ hybrid mice. The *Wdr76*^*−/−*^*; Apc*^*Min/+*^ mice exhibited increases in both the number and the size of tumors in the small intestine compared with 15-week-old age-matched *Wdr76*^*+/+*^*; Apc*^*Min/+*^ mice (Fig. 2d, e), indicating that Wdr76 plays a role as a tumor suppressor in the small intestine. In the *Wdr76*^*−/−*^*; Apc*^*Min/+*^ mice, the loss of *Wdr76* resulted in increased pan-Ras levels, especially in intestinal crypts and tumors, followed by increased proliferation of tumors, evidenced by increased numbers of PCNA-positive cells in crypts and tumors and a positive correlation between Ki67 expression and pan-Ras expression (Fig. 2f, g). Moreover, CSC activity was significantly increased in the tumors of the *Wdr76*^*−/−*^*; Apc*^*Min/+*^ mice compared with that in the tumors of *Wdr76*^*+/+*^*; Apc*^*Min/+*^ mice, as evidenced by increases in the levels of the CSC markers CD44, CD133 and CD166 (Fig. 2f). These results showed that the regulation of Ras protein stability by Wdr76 is important in CSC activity and tumorigenesis in CRC.

### Loss of WDR76 increases Wnt/β-catenin pathway activation in CRC

**Fig. 3:**
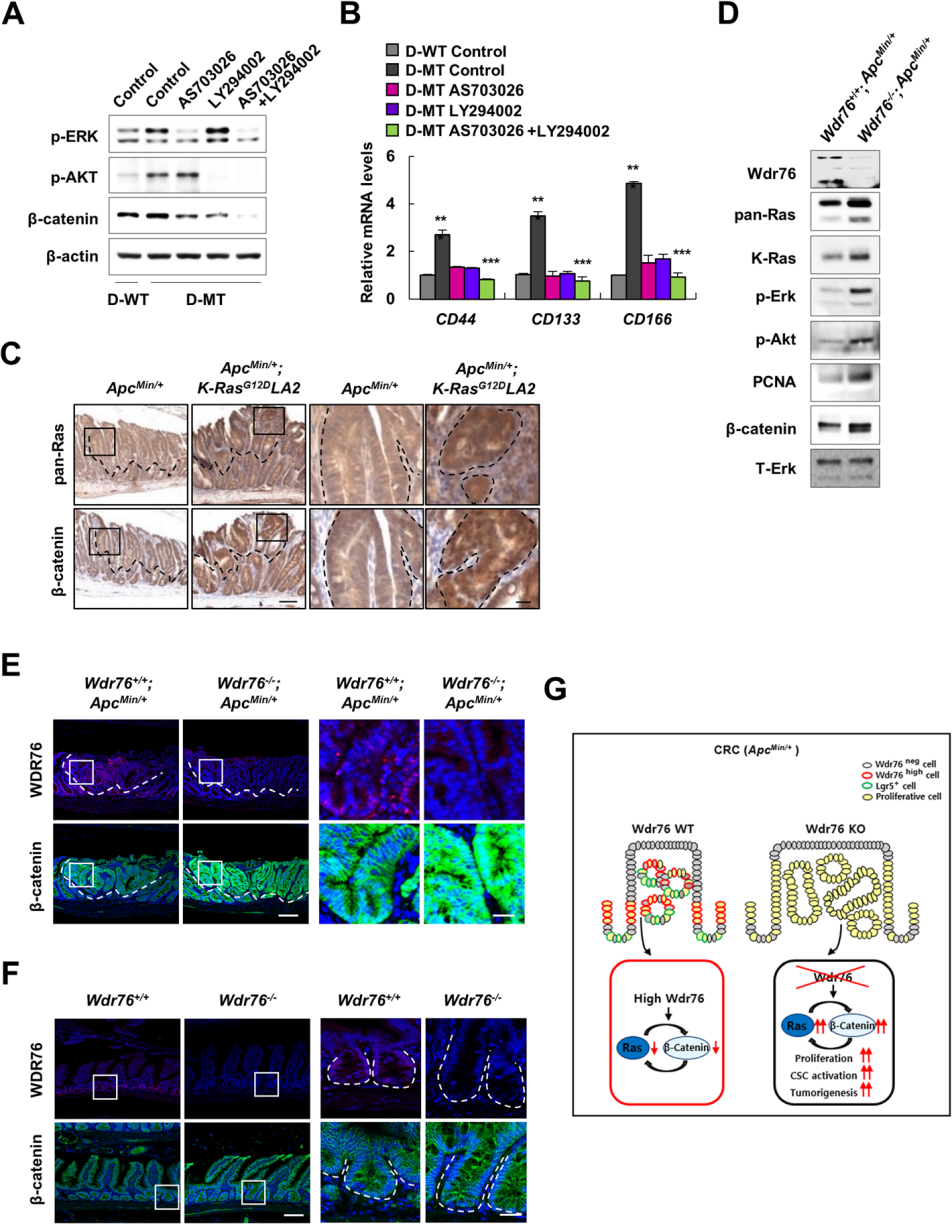
Loss of Wdr76 activates the Wnt/β-catenin pathway in CRC. **a** Western blot of D-WT and D-MT cells after treatment with AS703026 and LY294002. **b** Relative mRNA levels of CSC markers in D-WT and D-MT cells treated with AS703026 and LY294002. **c** Immunohistochemical staining of Ras and β-catenin in intestinal sections of 15-week-old *Apc*^*Min/+*^ and *Apc*^*Min/+*^*; K-Ras*^*G12D*^*LA2* mice. Boxes indicate the enlarged areas. Scale bars represent 100 μm (left panel) and 20 μm (right panel). **d** Western blots of tumors extracts from 15-week-old *Wdr76*^*+/+*^*; Apc*^*Min/+*^and *Wdr76*^*−/−*^*; Apc*^*Min/+*^ mice incubated with the indicated antibodies. **e** Confocal immunofluorescence of Wdr76 and β-catenin in small-intestine sections of 15-week-old *Wdr76*^*+/+*^*; Apc*^*Min/+*^ and *Wdr76*^*−/−*^*; Apc*^*Min/+*^ mice. Boxes indicate the enlarged areas. Tumors are indicated by dotted lines. T: Tumor. Scale bars represent 100 μm (left panel) and 20 μm (right panel). **f** Confocal immunofluorescence of Wdr76 and β-catenin in small-intestine sections of 15-week-old *Wdr76*^*+/+*^ and *Wdr76*^*−/−*^ mice. Boxes indicate the enlarged areas. Intestinal crypts are indicated by dotted lines. Scale bars represent 100 μm (left panel) and 20 μm (right panel). **g** Schema depicting the effects of *Wdr76* deletion in CRC harboring *Apc* mutation

In *APC*-mutated CRC, oncogenic *K-RAS* mutation enhances CSC activation and cooperatively promotes tumorigenesis by increasing Wnt/β-catenin pathway activation [15]. Wnt/β-catenin signaling is regulated via the MAPK/ERK and PI3K/AKT pathways, the major effector pathways downstream of RAS [15, 24, 25]. Consistent with previous findings, inhibition of the MAPK/ERK and PI3K/AKT pathways by the MEK inhibitor AS703026 and the PI3K inhibitor LY294002, respectively, reduced the β-catenin level, which had been increased by oncogenic K-RAS in D-MT cells harboring both *APC* and *K-RAS* mutations Co-treatment of both inhibitors further decreased the β-catenin level (Fig. 3a). Treatment with the MEK and PI3K inhibitors also suppressed oncogenic K-RAS-driven CSC activation in a manner that was correlated with the inhibition of the Wnt/β-catenin pathway, which plays pivotal roles in CSC activation (Fig. 3b). A strong positive correlation between β-catenin and pan-Ras expression levels in tumors of *Apc*^*Min/+*^*; K-Ras*^*G12D*^*LA2* mice (Fig. 3c) confirmed the importance of RAS stabilization in the positive crosstalk between the Wnt/β-catenin and RAS pathways in CRC harboring both *APC* mutations and oncogenic *K-RAS* mutations.

We next investigated whether Ras stabilization by loss of *Wdr76* affects the Wnt/β-catenin pathway. In lysates of tumors isolated from *Wdr76*^*+/+*^*; Apc*^*Min/+*^and *Wdr76*^*−/−*^*; Apc*^*Min/+*^ mice, the *Wdr76* knockout resulted in increased K-Ras and pan-Ras protein levels as well as increased Erk and Akt activities (Fig. 3d). The *Wdr76* deletion increased the β-catenin level in tumors of *Apc*^*Min/+*^ mice (Fig. 3d, e), but did not affect the β-catenin level in the absence of *Apc* mutation (Fig. 3f), showing that RAS stabilization activates the Wnt/β-catenin pathway in the presence of *Apc* mutation. These findings are consistent with previous reports that oncogenic *K-Ras* does not alter Wnt/β-catenin pathway activity by itself but does increase it in the presence of *Apc* mutation [13, 15].

Regulation of the Wnt/β-catenin pathway is important for Paneth cell differentiation [21, 26]. *Wdr76* deficiency not only enriched CSCs in the tumors of *Apc*^*Min/+*^ mice but also increased the numbers of Paneth cells, which provide the niche for CSC maintenance, in both crypts and tumors of *Apc*^*Min/+*^ mice (Additional file 1: Figure S2C). Paneth cell differentiation was not affected by *Wdr76* deletion alone, correlating with no changes in the Wnt/β-catenin pathway regulation by *Wdr76* deletion (Additional file 1: Figure S2B). Consistent with those results in *Wdr76*^*−/−*^ mice, goblet cell differentiation was also increased in the tumors of *Wdr76*^*−/−*^*; Apc*^*Min/+*^ mice (Additional file 1: Figure S2C).

Together, our results indicated that in *APC*-mutated CRC tumors, WDR76 plays a role as a tumor suppressor by repressing RAS protein abundance and thus reducing the activation of the Wnt/β-catenin pathway. Thus, WDR76 deletion in CRC further increases the RAS protein level and the crosstalk between the RAS and Wnt/β-catenin pathways, resulting in increased proliferation, CSC activation and tumorigenesis (Fig. 3g).

### WDR76 reduces RAS levels through polyubiquitination-dependent proteasomal degradation in CRC

**Fig. 4:**
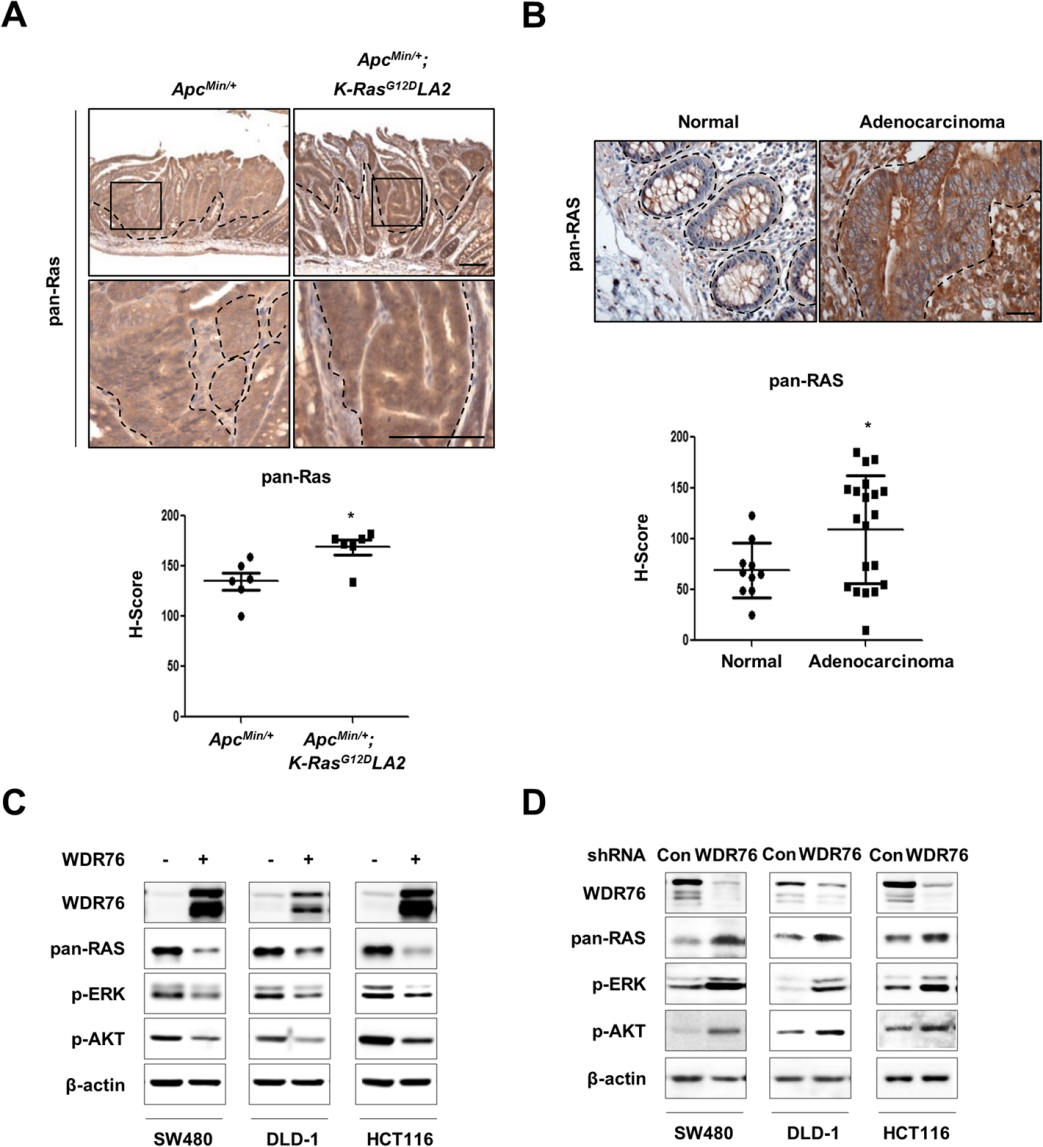
WDR76 regulates the RAS protein level and downstream signaling pathway activities in CRC cells. **a** Immunohistochemical staining of Ras in intestinal sections of *Apc*^*Min/+*^and *Apc*^*Min/+*^*; K-Ras*^*G12D*^*LA2* mice and quantifications of Ras expression using Image J. * *p* < 0.05. **b** Immunohistochemical staining of RAS in intestinal sections of from human patients with CRC microarray and quantification of RAS expression using Image J. * *p* < 0.05. **a-b** Western blot of extracts from CRC cell lines SW480, DLD-1, and HCT116 transiently transfected with Flag-Control (Control) or Flag-WDR76 (WDR76 OE) **a** and shControl-GFP (Control) or shWDR76-GFP (WDR76 KD) **b** using the indicated antibodies

Oncogenic *K-Ras* mutation involving tumor progression further increased the Ras protein level in *Apc*^*Min/+*^*; K-Ras*^*G12D*^*LA2* mouse tumors compared with that in *Apc*^*Min/+*^ mouse tumors (Fig. 4a). Furthermore, pan-RAS protein levels were increased in patients with colon adenocarcinoma (Fig. 4b). Based on the previous finding which identified that WDR76 destabilizes all three major isoforms of RAS and suppresses HCC tumorigenesis [19], the effect of WDR76 on RAS protein levels has been tested in CRC cell lines with various genetic backgrounds. As in HCC, overexpression and knockdown of WDR76 in CRC cell lines decreased and increased pan-RAS protein levels, respectively, resulting in the regulation of the activities of its downstream effectors ERK and AKT (Fig. 4c, d).

**Fig. 5:**
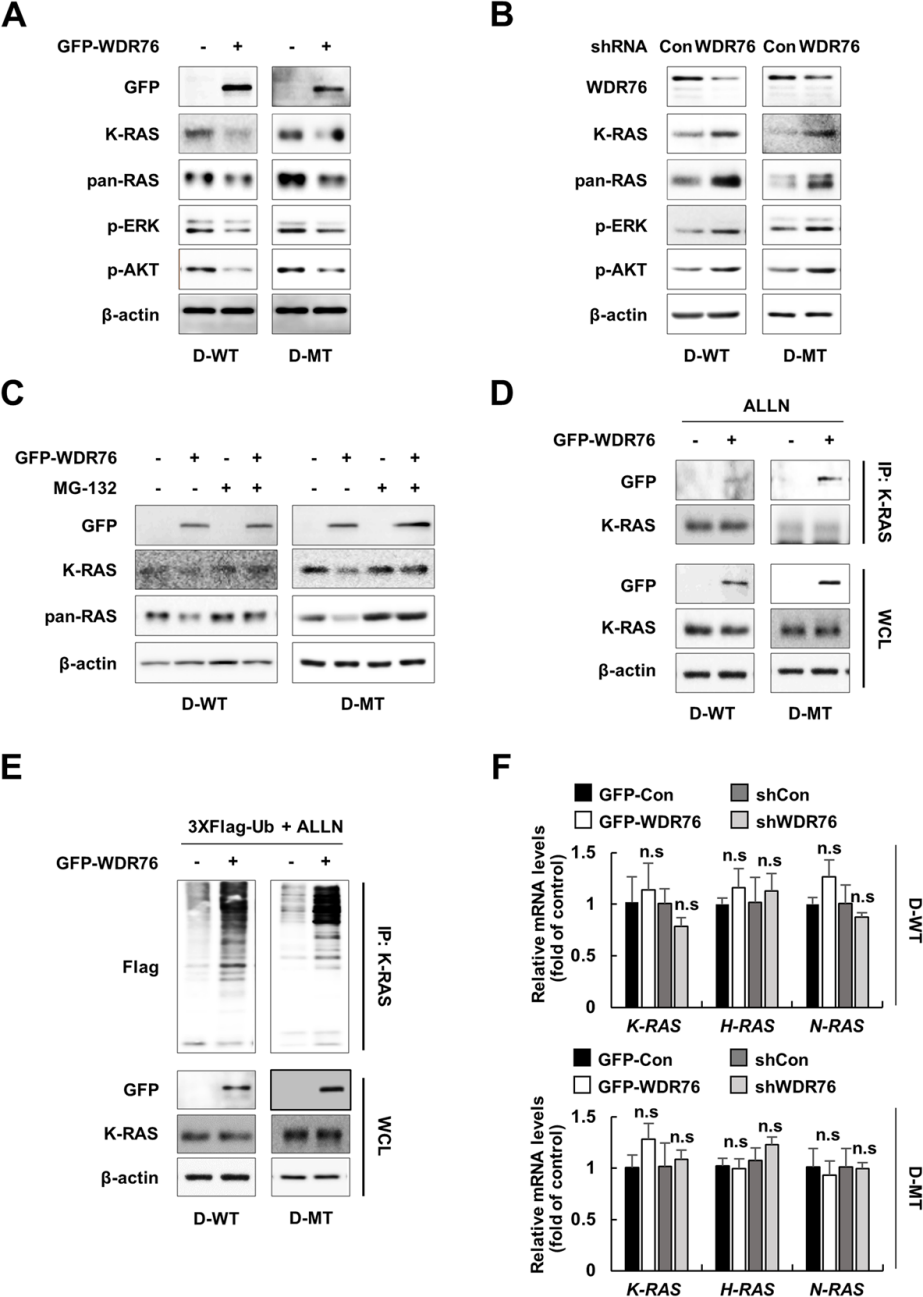
WDR76 induces proteasomal degradation of wild-type and oncogenic K-RAS in CRC cells. **a-b** Western blots of extracts from D-WT and D-MT cells stably expressing GFP-Control or GFP-WDR76 **a** and shControl-GFP or shWDR76-GFP **b** using the indicated antibodies. **c** Western blots of extracts from D-WT and D-MT cells stably expressing GFP-Control or GFP-WDR76 with or without MG-132 treatment (20 μM, 4 h) using the indicated antibodies. **d-e** Immunoprecipitation **d** and ubiquitination **e** of K-RAS in ALLN-treated (25 μg/mL, 12 h) D-WT and D-MT cells stably expressing GFP-Control or GFP-WDR76 with immunoblotting against the indicated antibodies. **f** Relative mRNA levels of the indicated genes from D-WT and D-MT cells stably expressing either GFP-Control, GFP-WDR76, shControl-GFP, or shWDR76-GFP quantified by RT-qPCR. n.s.: not significant

Since 50% of CRC patients harbor oncogenic *K-RAS* mutations, we investigated whether WDR76 regulates the stability of wild-type and oncogenic forms of K-RAS in CRC. We examined the effects of WDR76 overexpression on steady-state levels of K-RAS using *APC*-mutant D-WT and D-MT cells, which are isogenic except that they harbor wild-type and mutant *K-RAS*, respectively [15]. WDR76 overexpression significantly decreased the levels of both wild-type and oncogenic K-RAS and consequently inhibited the activities of ERK and AKT (Fig. 5a). Accordingly, WDR76 knockdown markedly increased endogenous K-RAS and pan-RAS protein levels, resulting in the activation of ERK and AKT (Fig. 5b). In addition, the proteasomal inhibitor MG-132 could reverse the WDR76 overexpression-induced reduction of the K-RAS level (Fig. 5c), indicating that WDR76 degrades K-RAS via the proteasomal pathway. The interaction of WDR76 with both wild-type and oncogenic K-RAS was confirmed in D-WT and D-MT cells, respectively (Fig. 5d). Next, we examined whether WDR76 could regulate ubiquitination of K-RAS. WDR76 overexpression markedly enhanced the polyubiquitination of K-RAS (Fig. 5e), confirming that K-RAS is a substrate of WDR76. There were no changes in the mRNA levels of *K-RAS*, *H-RAS*, and *N-RAS*, the major isoforms of RAS (Fig. 5f), which confirmed that the regulation of RAS by WDR76 was not a result of transcriptional regulation.

### WDR76 suppresses CSC activation in spheroids of CRC cells harboring oncogenic *KRAS*

**Fig. 6:**
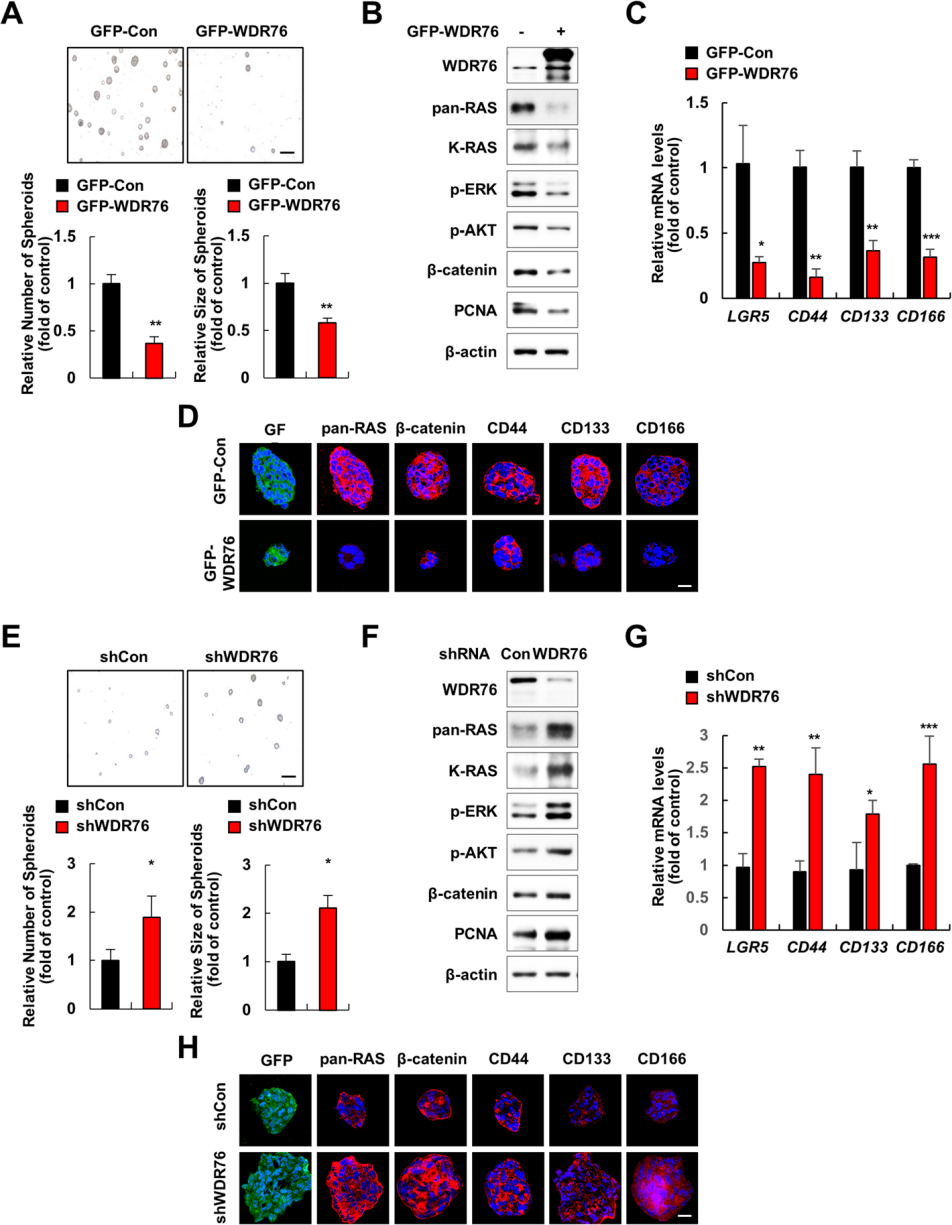
WDR76 regulates CSC activation in CRC spheroids harboring oncogenic *KRAS* mutation. **a-d** Five-day spheroid cultures of D-MT cells stably expressing GFP-Control or GFP-WDR76 were analyzed. **a** Number and size of spheroids were quantified using Image J. *** *p* < 0.001. * *p* < 0.05, ** *p* < 0.01. **b** Extracts were immunoblotted using the indicated antibodies. **c** Relative mRNA levels of the indicated genes were quantified by RT-qPCR. * *p* < 0.05, ** *p* < 0.01, and *** *p* < 0.001. **d** Immunocytochemistry was performed using the indicated antibodies and counterstaining with DAPI. Scale bars represent 20 μm. **e-h** Five-day spheroid cultures of D-MT cells stably expressing shControl-GFP or shWDR76-GFP were analyzed. **e** Number and size of spheroids were quantified using Image J. * *p* < 0.05, ** *p* < 0.01. **f** Extracts were immunoblotted using the indicated antibodies. **g** Relative mRNA levels of the indicated genes were quantified by RT-qPCR. * *p* < 0.05, ** *p* < 0.01, and *** *p* < 0.001. **h** Immunocytochemistry was performed using the indicated antibodies and counterstaining with DAPI. Scale bars represent 20 μm

Oncogenic K-RAS plays crucial roles in CSC activation [15, 27]. We sorted D-MT cells based on expression levels of the CSC markers CD44, CD133, and CD166. The cells with high expression of the CSC markers had higher pan-RAS protein levels and increased downstream effector pathway activities than the cells with low expression of the CSC markers (Additional file 1: Figure S3A, B). We next examined whether WDR76-mediated destabilization of RAS suppresses CSC properties. D-MT cells with stable GFP-WDR76 overexpression showed diminished sphere-forming ability (Fig. 6a) along with decreased K-RAS and pan-RAS levels, decreased ERK and AKT activities, and decreased β-catenin levels (Fig. 6b). Consistent with the effects on sphere-forming ability, WDR76 overexpression significantly reduced the mRNA levels of the CSC markers *LGR5*, *CD44*, *CD133* and *CD166* (Fig. 6c). Immunocytochemical analyses confirmed the WDR76 overexpression-mediated decrease of pan-RAS levels followed by the reduction of β-catenin and CSC markers in the D-MT spheroids (Fig. 6d). On the other hand, WDR76 knockdown by shRNA increased the sphere-forming ability of D-MT cells (Fig. 6e) and resulted in increased K-RAS and pan-RAS levels, increased ERK and AKT activities, and increased β-catenin levels (Fig. 6f). The shWDR76 sphere cells displayed increased mRNA levels of the CSC markers *LGR5*, *CD44*, *CD133* and *CD166* (Fig. 6g). Immunocytochemical analyses confirmed the WDR76 knockdown-mediated stabilization of pan-RAS followed by the increase in β-catenin and CSC markers in the D-MT spheroids (Fig. 6h).

### Cytosolic WDR76 destabilizes RAS and suppresses oncogenic *K-RAS*-driven CSC activation

To identify whether a certain subcellular fraction of WDR76 is involved in K-RAS destabilization in CRC, we overexpressed full-length GFP-WDR76 and a nuclear localization signal (NLS)-deleted mutant of GFP-WDR76 (FL and ΔNLS, respectively) in D-MT cells. Consistent with the previous study demonstrating that cytosolic WDR76 is involved in the destabilization of H-RAS and the suppression of transformation in HCC [19], GFP-WDR76ΔNLS was more effective than GFP-WDR76FL in reducing K-RAS and pan-RAS protein levels (Additional file 1: Figure S4A). Overexpression of cytosolic WDR76 increased the amount of WDR76 bound to K-RAS and the polyubiquitination of K-RAS (Additional file 1: Figure S4B, C). Accordingly, the NLS-deleted mutant WDR76 more effectively inhibited sphere formation (Additional file 1: Figure S4D), growth (Additional file 1: Figure S4E) and CSC activation than the full-length WDR76, as evidenced by mRNA levels of CSC markers (Additional file 1: Figure S4F, G), indicating that cytosolic WDR76 destabilizes RAS and suppresses CSC activation.

## Discussion

Targeting oncogenic K-RAS involving progression of colorectal adenoma to adenocarcinoma still remains as an urgent and unmet therapeutic need [28]. Although approximately 90% of CRCs harbor *APC* mutations as the initiating events leading to clonal expansion of colonic stem cells and adenoma formation, the dysregulation of β-catenin and RAS is not sufficient to generate the CRC phenotype. The progression to adenocarcinoma requires additional oncogenic *K-RAS* mutations, which lead to further clonal expansion of the CSC population via further enhancement of Wnt/β-catenin signaling. During that process, the stabilization of RAS caused by *APC* mutations plays important roles in the amplification of Wnt/β-catenin and RAS signaling and cancer progression via activation of the ERK and PI3K/AKT pathways downstream of RAS [15, 20, 29]. These results suggest RAS destabilization as a pathophysiologic strategy to overcome the limitations of treating oncogenic K-RAS-driven CRC [16]. The recent identification of a small molecule that degrades RAS independently of the Wnt/β-catenin pathway and suppresses the transformation and proliferation of CRC cells harboring non-degradable β-catenin indicates the existence of an alternative way for RAS stability regulation which plays important roles in CRC tumorigenesis [18]. Direct degradation of RAS to suppress oncogenic K-RAS-mediated enhancement of CSC activation may provide a novel approach to selectively target CSCs that contribute to cancer progression.

We found the role of WDR76, a newly identified RAS-binding protein that mediates RAS degradation [19], in the suppression of CRC, especially that related with the *K-RAS* mutation-induced activation of CSCs. WDR76 is mostly expressed in the intestinal crypt side walls, where it restrains the accumulation of RAS, thus restricting the proliferation of crypt cells and helping to maintain intestinal homeostasis. The inverse correlation between WDR76 expression and LGR5 expression in murine and human CRCs emphasizes the pathological importance of WDR76 destabilizing RAS as a tumor suppressor inhibiting CSC activities. WDR76 caused the ubiquitination-dependent proteasomal degradation of both wild-type and oncogenic K-RAS and had suppressive effects on cancer stemness in CRC cell line-derived spheroids harboring both *APC* and *K-RAS* mutations. NLS-deleted WDR76 caused increased degradation of K-RAS and increased suppression of CSCs compared with wild-type WDR76, indicating that the RAS-degrading and CSC-suppressing effects of WDR76 are caused by cytosolic, rather than nuclear, WDR76. Depletion of Wdr76 exacerbated the tumorigenic phenotypes of *Apc*^*Min/+*^ mice and caused significant increases in Ras protein levels and CSC marker levels, suggesting that the CSC activation and cancer-promoting effect are controlled not only by the occurrence of oncogenic RAS mutation but also by the RAS stabilization.

The Wnt/β-catenin pathway is important in CRC initiation, but it also plays a pivotal role in the sustenance of ISCs. Because of that, targeting Wnt/β-catenin signaling results in intestinal toxicity [30]. On the other hand, RAS signaling is important in the proliferation of *LGR5*^*+*^ ISCs but not in the maintenance of normal ISCs [13, 21]. In the absence of *Apc* mutation, Ras stabilization via *Wdr76* deletion did not affect the Wnt/β-catenin pathway.

## Conclusions

Our study suggests that by targeting the stability of the RAS protein, it is possible to suppress the downstream signaling activities of RAS and reduce the activation of Wnt/β-catenin signaling involving CSC activation. The reduction of elevated RAS protein levels caused by *APC* and *KRAS* mutations might be an ideal therapeutic strategy that can target both CSCs and non-CSC cancer cells that have the potential to dedifferentiate into CSCs, without causing toxicity to healthy stem cells. Our results suggest that the regulation of RAS stability by WDR76 is a potential strategy for targeting malignant CRC involving CSC activation.

## Additional file

Additional file 1: **Figure S1.** Ras protein level is increased in Lgr5^+^ stem cells in the murine small intestine. A-B Immunofluorescence of **A** Ras (green) and **B** Lgr5^-^ GFP (green) in *Lgr5-EGFP* mouse intestinal sections. Lgr5^+^ ISCs are indicated by arrows. Scale bars represent 20 μm. **Figure S2.** Loss of Wdr76 affects lineage differentiation in the murine small intestine. **A** Quantification of the length of small intestinal crypts, based on at least 10 crypts per 5 fields of view. *** *p* < 0.001. **B-C** Immunofluorescence analysis of lineage differentiation into goblet cells (mucin2, red) and Paneth cell (lysozyme, red) in intestinal sections of **B** *Wdr76*^*+/+*^ and *Wdr76*^*-/-*^ mice and **C** *Wdr76*^*+/+*^; *Apc*^*Min/+*^ and *Wdr76*^*−/−*^; *Apc*^*Min/+*^ mice. Boxes indicate the enlarged areas. **B** Crypts and **C** tumors are indicated by dotted lines. Scale bars represent 20 μm. **Figure S3.** RAS protein level is increased in CSC-like cells compared with that in non-CSC-like cells in CRC. **A-B** Non-CSC-like cells (CD44^low^CD133^-^CD166^-^) and CSC-like cells (CD44^high^CD133^+^CD166^+^ cells) were sorted from D-MT cells by flow cytometry and were analyzed by **A** brightfield images of spheroid cultures and **B** western blots using the indicated antibodies. **A** Scale bars represent 20 μm. **Figure S4.** Cytosolic WDR76 destabilizes RAS and suppresses CSC activation in CRC. **A-G** D-MT cells stably expressing GFP-Control, GFP-WDR76FL, or GFP-WDR76ΔNLS were analyzed. **A** Western blots using the indicated antibodies. **B-C** After treatment of ALLN (25 μg/mL, 12 h), extracts were analyzed by **B** immunoprecipitation and **C** ubiquitination of K-RAS by immunoblotting against the indicated antibodies. **D-G** Five-day spheroid cultures were analyzed. **D** Number and size of spheroids were quantified using Image J. *** *p* < 0.001. **E** Cell viability assay was performed. *** *p* < 0.001. **F** Immunocytochemistry was performed using the indicated antibodies and counterstaining with DAPI. Scale bars represent 20 μm. **G** Relative mRNA levels of the indicated genes were quantified by RT-qPCR. * *p* < 0.05, ** *p* < 0.01. (DOCX 3962 kb)

## Abbreviations

APC: Adenomatous polyposis coli
CRC: Colorectal cancer
CSC: Cancer stem cell
EGF: Epidermal growth factor
EGFR: Epidermal growth factor receptor
HCC: Hepatocellular carcinoma
ISC: Intestinal stem cell
MAPK: Mitogen-activated protein kinase
NLS: Nuclear localization signal
TA: Transit amplifying
WDR76: WD Repeat protein 76

## References

[1] Barker N, Ridgway RA, van Es JH, van de Wetering M, Begthel H, van den Born M (2009). Crypt stem cells as the cells-of-origin of intestinal cancer. Nature..

[2] Merlos-Suarez A, Barriga FM, Jung P, Iglesias M, Cespedes MV, Rossell D (2011). The intestinal stem cell signature identifies colorectal cancer stem cells and predicts disease relapse. Cell Stem Cell.

[3] Zeuner A, De Maria R (2011). Not so lonely at the top for cancer stem cells. Cell Stem Cell.

[4] Ricci-Vitiani L, Lombardi DG, Pilozzi E, Biffoni M, Todaro M, Peschle C (2007). Identification and expansion of human colon-cancer-initiating cells. Nature..

[5] Plaks V, Kong N, Werb Z (2015). The cancer stem cell niche: how essential is the niche in regulating stemness of tumor cells?. Cell Stem Cell.

[6] Junttila MR, Mao W, Wang X, Wang BE, Pham T, Flygare J (2015). Targeting LGR5+ cells with an antibody-drug conjugate for the treatment of colon cancer. Sci Transl Med.

[7] Vidal SJ, Rodriguez-Bravo V, Galsky M, Cordon-Cardo C, Domingo-Domenech J (2014). Targeting cancer stem cells to suppress acquired chemotherapy resistance. Oncogene..

[8] Fevr T, Robine S, Louvard D, Huelsken J (2007). Wnt/beta-catenin is essential for intestinal homeostasis and maintenance of intestinal stem cells. Mol Cell Biol.

[9] Fujii M, Shimokawa M, Date S, Takano A, Matano M, Nanki K (2016). A colorectal tumor organoid library demonstrates progressive loss of niche factor requirements during tumorigenesis. Cell Stem Cell.

[10] Bos JL, Fearon ER, Hamilton SR, Verlaan-de Vries M, van Boom JH, van der Eb AJ (1987). Prevalence of ras gene mutations in human colorectal cancers. Nature..

[11] Chung DC (2000). The genetic basis of colorectal cancer: insights into critical pathways of tumorigenesis. Gastroenterology..

[12] Fearon ER, Vogelstein B (1990). A genetic model for colorectal tumorigenesis. Cell..

[13] Feng Y, Bommer GT, Zhao J, Green M, Sands E, Zhai Y (2011). Mutant KRAS promotes hyperplasia and alters differentiation in the colon epithelium but does not expand the presumptive stem cell pool. Gastroenterology.

[14] Snippert HJ, Schepers AG, van Es JH, Simons BD, Clevers H (2014). Biased competition between Lgr5 intestinal stem cells driven by oncogenic mutation induces clonal expansion. EMBO Rep.

[15] Moon BS, Jeong WJ, Park J, Kim TI, Min do S, Choi KY. Role of oncogenic K-Ras in cancer stem cell activation by aberrant Wnt/beta-catenin signaling. J Natl Cancer Inst. 2014;106(2):djt373. 10.1093/jnci/djt373 24491301

[16] Jeong WJ, Yoon J, Park JC, Lee SH, Lee SH, Kaduwal S (2012). Ras stabilization through aberrant activation of Wnt/beta-catenin signaling promotes intestinal tumorigenesis. Sci Signal.

[17] Lee SK, Jeong WJ, Cho YH, Cha PH, Yoon JS, Ro EJ, et al. beta-Catenin-RAS interaction serves as a molecular switch for RAS degradation via GSK3beta. EMBO Rep. 2018;19(12):e46060. 10.15252/embr.201846060 PMC6280641 30413483

[18] Shin W, Lee SK, Hwang JH, Park JC, Cho YH, Ro EJ (2018). Identification of Ras-degrading small molecules that inhibit the transformation of colorectal cancer cells independent of beta-catenin signaling. Exp Mol Med.

[19] Jeong WJ, Park JC, Kim WS, Ro EJ, Jeon SH, Lee SK (2019). WDR76 is a RAS binding protein that functions as a tumor suppressor via RAS degradation. Nat Commun.

[20] Cho YH, Cha PH, Kaduwal S, Park JC, Lee SK, Yoon JS (2016). KY1022, a small molecule destabilizing Ras via targeting the Wnt/beta-catenin pathway, inhibits development of metastatic colorectal cancer. Oncotarget..

[21] Basak O, Beumer J, Wiebrands K, Seno H, van Oudenaarden A, Clevers H (2017). Induced quiescence of Lgr5+ stem cells in intestinal organoids enables differentiation of hormone-producing Enteroendocrine cells. Cell Stem Cell.

[22] Chen Q, Zhang X, Li WM, Ji YQ, Cao HZ, Zheng P (2014). Prognostic value of LGR5 in colorectal cancer: a meta-analysis. PLoS One.

[23] Su LK, Kinzler KW, Vogelstein B, Preisinger AC, Moser AR, Luongo C (1992). Multiple intestinal neoplasia caused by a mutation in the murine homolog of the APC gene. Science..

[24] Fearon ER, Wicha MS (2014). KRAS and cancer stem cells in APC-mutant colorectal cancer. J Natl Cancer Inst.

[25] Li J, Mizukami Y, Zhang X, Jo WS, Chung DC (2005). Oncogenic K-ras stimulates Wnt signaling in colon cancer through inhibition of GSK-3beta. Gastroenterology..

[26] van Es JH, Jay P, Gregorieff A, van Gijn ME, Jonkheer S, Hatzis P (2005). Wnt signalling induces maturation of Paneth cells in intestinal crypts. Nat Cell Biol.

[27] Cha ST, Tan CT, Chang CC, Chu CY, Lee WJ, Lin BZ (2016). G9a/RelB regulates self-renewal and function of colon-cancer-initiating cells by silencing let-7b and activating the K-RAS/beta-catenin pathway. Nat Cell Biol.

[28] Stephen AG, Esposito D, Bagni RK, McCormick F (2014). Dragging ras back in the ring. Cancer Cell.

[29] Cha PH, Cho YH, Lee SK, Lee J, Jeong WJ, Moon BS (2016). Small-molecule binding of the axin RGS domain promotes beta-catenin and Ras degradation. Nat Chem Biol.

[30] Zhong Y, Katavolos P, Nguyen T, Lau T, Boggs J, Sambrone A (2016). Tankyrase inhibition causes reversible intestinal toxicity in mice with a therapeutic index < 1. Toxicol Pathol.

