# Supplementary material for: WDR76 degrades RAS and suppresses cancer stem cell activation in colorectal cancer
Source: Cell Commun Signal. 2019 Jul 30;17:88. doi: 10.1186/s12964-019-0403-x (PMC6668196; doi:10.1186/s12964-019-0403-x)
Supplement: Supplementary file 1 — Figure S1. Ras protein level is increased in Lgr5+ stem cells in the murine small intestine. A-B Immunofluorescence of A Ras (green) and B Lgr5- GFP (green) in Lgr5-EGFP mouse intestinal sections. Lgr5+ ISCs are indicated by arrows. Scale bars represent 20 μm. Figure S2. Loss of Wdr76 affects lineage differentiation in the murine small intestine. A Quantification of the length of small intestinal crypts, based on at least 10 crypts per 5 fields of view. *** p < 0.001. B-C Immunofluorescence analysis of lineage differentiation into goblet cells (mucin2, red) and Paneth cell (lysozyme, red) in intestinal sections of B Wdr76+/+ and Wdr76-/- mice and C Wdr76+/+; ApcMin/+ and Wdr76−/−; ApcMin/+ mice. Boxes indicate the enlarged areas. B Crypts and C tumors are indicated by dotted lines. Scale bars represent 20 μm. Figure S3. RAS protein level is increased in CSC-like cells compared with that in non-CSC-like cells in CRC. A-B Non-CSC-like cells (CD44lowCD133-CD166-) and CSC-like cells (CD44highCD133+CD166+ cells) were sorted from D-MT cells by flow cytometry and were analyzed by A brightfield images of spheroid cultures and B western blots using the indicated antibodies. A Scale bars represent 20 μm. Figure S4. Cytosolic WDR76 destabilizes RAS and suppresses CSC activation in CRC. A-G D-MT cells stably expressing GFP-Control, GFP-WDR76FL, or GFP-WDR76ΔNLS were analyzed. A Western blots using the indicated antibodies. B-C After treatment of ALLN (25 μg/mL, 12 h), extracts were analyzed by B immunoprecipitation and C ubiquitination of K-RAS by immunoblotting against the indicated antibodies. D-G Five-day spheroid cultures were analyzed. D Number and size of spheroids were quantified using Image J. *** p < 0.001. E Cell viability assay was performed. *** p < 0.001. F Immunocytochemistry was performed using the indicated antibodies and counterstaining with DAPI. Scale bars represent 20 μm. G Relative mRNA levels of the indicated genes were quantified by RT-qPCR. * p < 0.05, * [file 12964_2019_403_MOESM1_ESM.docx]

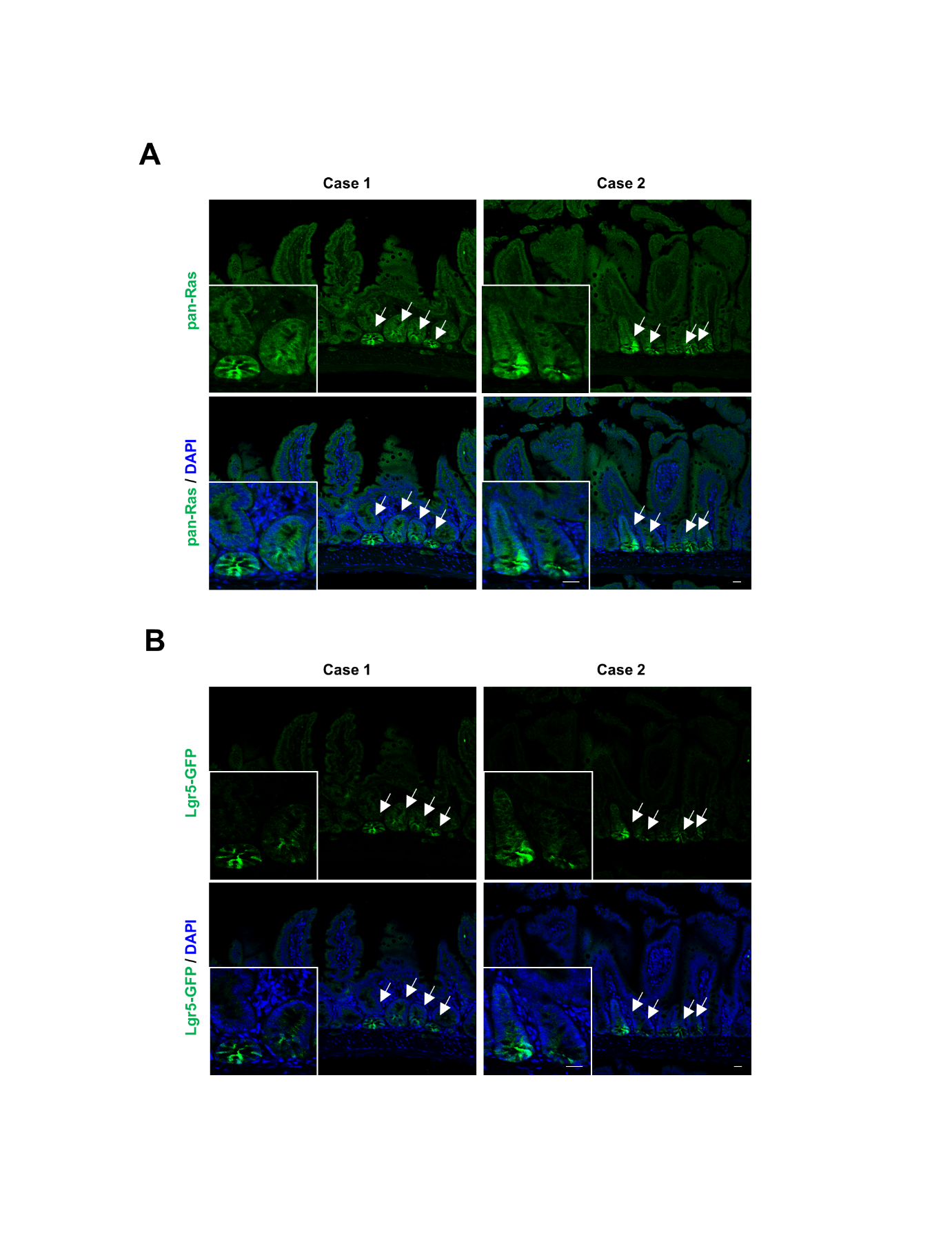


**Figure S1** The Ras protein level is increased in Lgr5^+^ stem cells in the murine small intestine. **A-B** Confocal immunofluorescence of **A** Ras (green) and **B** Lgr5-GFP (green) in *Lgr5- EGFP* mouse intestinal sections. Lgr5^+^ ISCs indicated by arrows. Scale bars represent 20 μm.


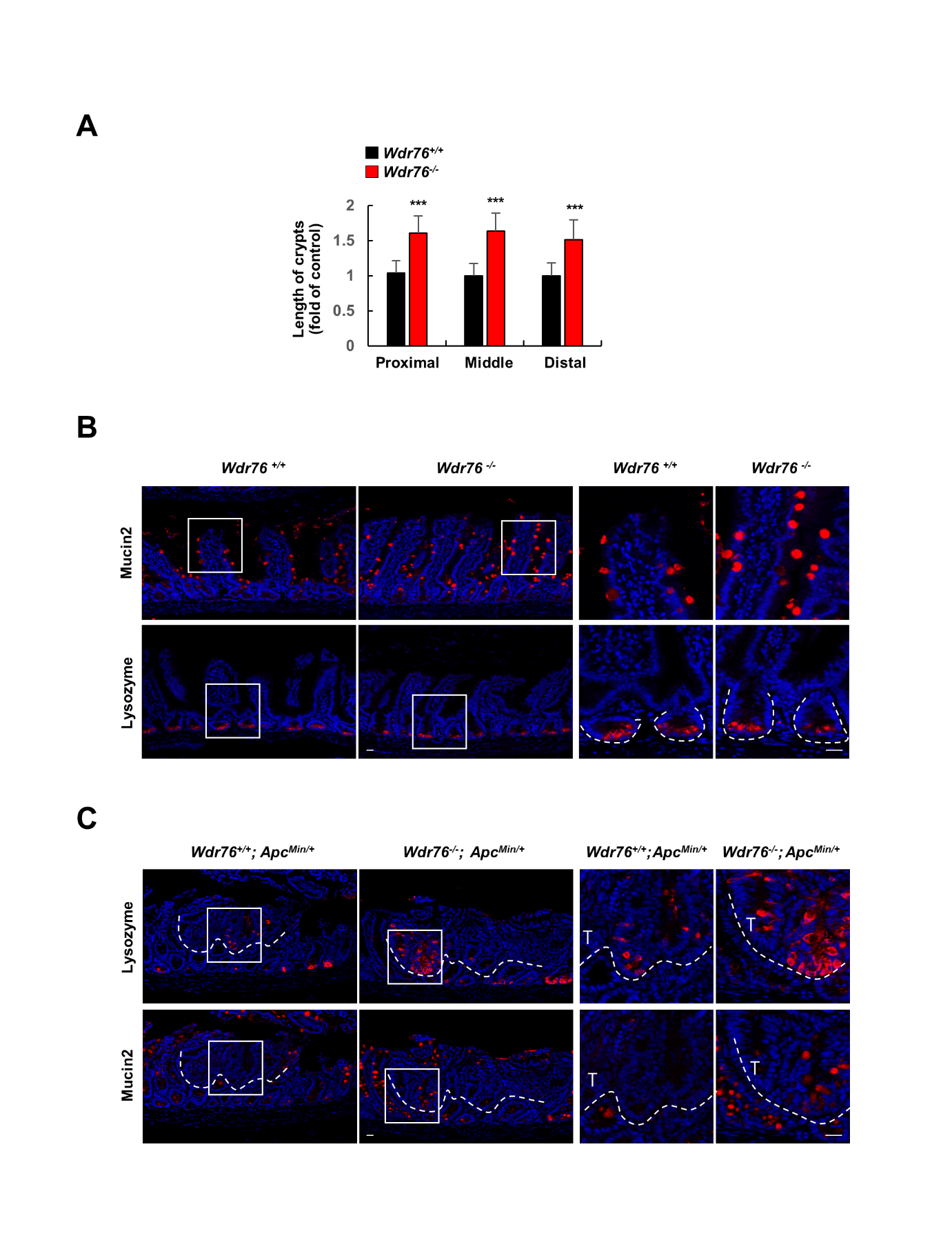


**Figure S2** Loss of Wdr76 affects lineage differentiation in the murine small intestine. **A** Quantification of the length of small intestinal crypts. All measurements or counts are based on at least 10 crypts per 5 fields of view. *** p<0.001. **B-C** Comparative confocal immunofluorescence analysis of lineage differentiation into goblet cells (mucin2, red) and Paneth cell (lysozyme, red) in intestinal sections of **B** *Wdr76^+/+^* and *Wdr76^-/-^* mice and **C** *Wdr76^+/+^*; *Apc^Min/+^* and *Wdr76^-/-^*; *Apc^Min/+^* mice. Boxes indicate the enlarged areas. **B** Crypts and **C** tumors are indicated by dotted lines. Scale bars represent 20 μm.


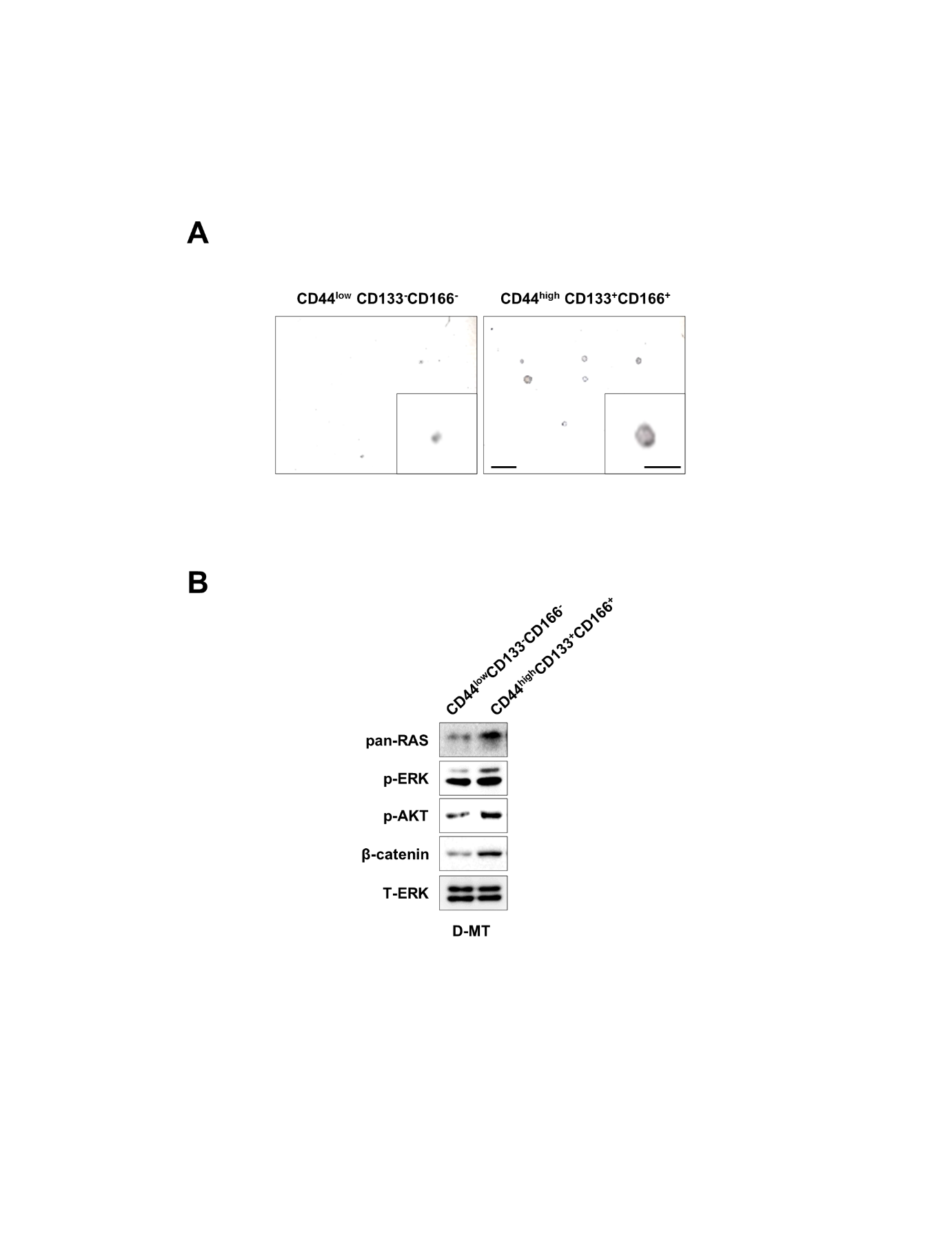


**Figure S3** RAS protein level is increased in CSC-like cells compared with that in non-CSC-like cells in CRC. **A** Brightfield images of spheroid cultures of CD44^low^CD133^-^CD166^-^ and CD44^high^CD133^+^CD166^+^ cells sorted from D-MT cells by flow cytometry for the indicated antibodies. Scale bars represent 20 μm. **B** Western blots of extracts from CD44^low^CD133^-^CD166^-^ and CD44^high^CD133^+^CD166^+^ cells sorted from D-MT cells using the indicated antibodies.


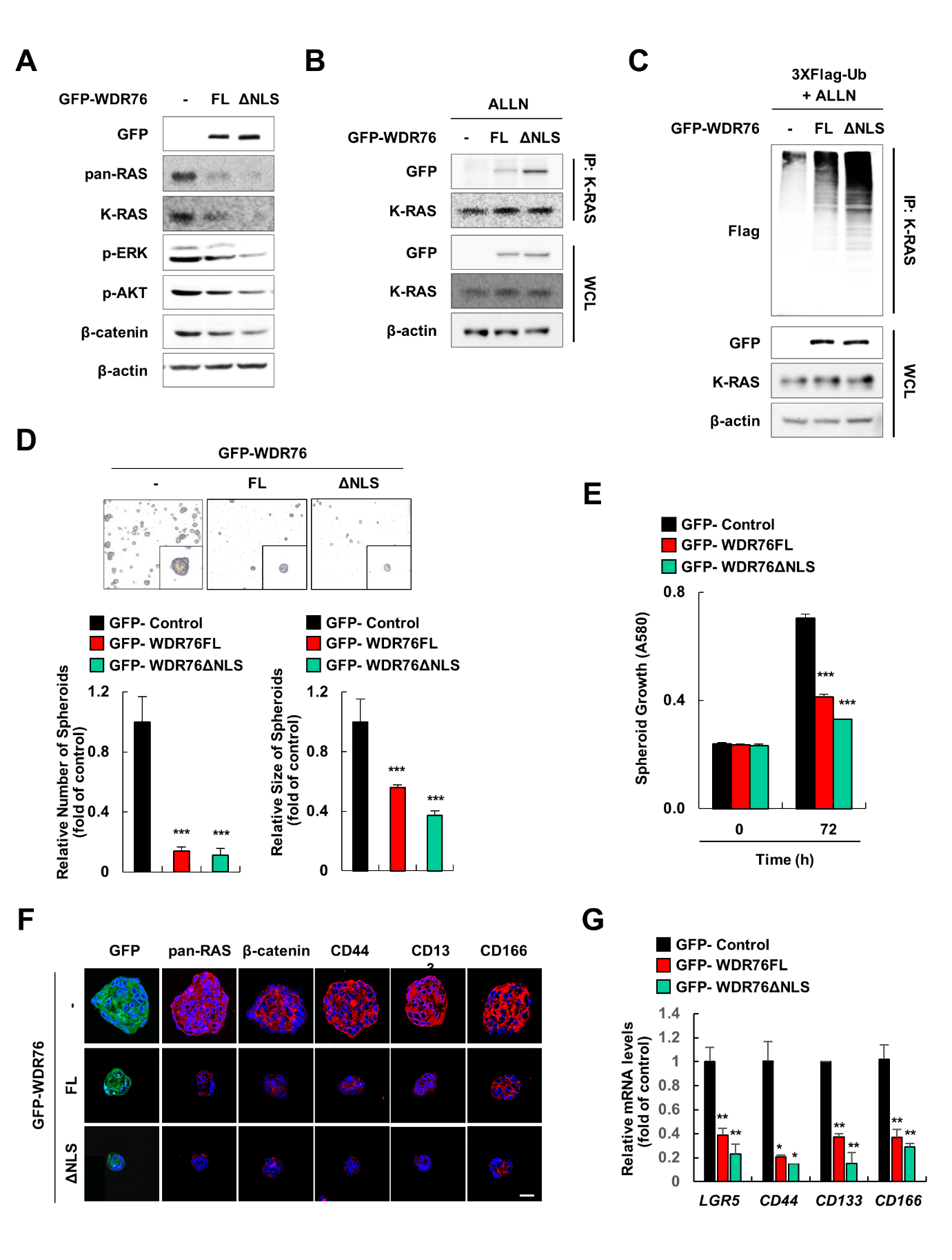


**Figure S4** Cytosolic WDR76 destabilizes RAS and suppresses CSC activation in CRC. **A** Western blots of extracts from D-MT cells stably expressing GFP-Control, GFP-WDR76FL, or GFP-WDR76ΔNLS using the indicated antibodies. **B-C** Immunoprecipitation **B** and ubiquitination **C** of K-RAS in ALLN-treated (25 μg/mL, 12 h) D-MT cells stably expressing GFP-Control, GFP-WDR76FL, or GFP-WDR76ΔNLS with immunoblotting against the indicated antibodies. **D-G** Five-day spheroid cultures of D-MT cells stably expressing GFP-Control, GFP-WDR76FL, or GFP-WDR76ΔNLS were analyzed. **D** Number and size of spheroids were quantified using Image J. *** p<0.001. **E** Cell viability assay was performed at the indicated culture day. *** p<0.001. **F** Immunocytochemistry was performed using the indicated antibodies and counterstaining with DAPI. Scale bars represent 20 μm. **G** Relative mRNA levels of the indicated genes were quantified by RT-qPCR. * p<0.05, ** p<0.01.
